# Supplementary material for: WordCommentsAnalyzer: A windows software tool for qualitative research
Source: F1000Res. 2018 Sep 4;7:536. Originally published 2018 May 3. [Version 2] doi: 10.12688/f1000research.14819.2 (PMC6137411; doi:10.12688/f1000research.14819.2)
Supplement: Supplementary file 3 [file f1000research-7-17614-s0002.tgz › b2a79496-4ada-47c4-9682-a99ce867836d.zip!/Eight Iranian Health Journals/Archives of Rehabilitation_72.docx]

© Archives of Rehabilitation. This work is licensed under CC BY-NC 4.0 (https://creativecommons.org/licenses/by-nc/4.0/).

Volume & Issue: دوره 1 - شماره 2

Title: بررسی تاثیر تمرین ذهنی روی هماهنگی حرکتی اندام فوقانی در بیماران همی پلژیک

http://rehabilitationj.uswr.ac.ir/article-1-511-fa.html

هدف: تمرین ذهنی به عنوان بازسازی یک الگوی حرکتی در ذهن مطرح است وجود عواملی نظیر تشابه زمانی بین اجرای فیزیک یک فعالیت و اجرای ذهنی آن، افزایش جریان خون مغزی ، افزایش فعالی سیستم اتونوم در حین تمرین ذهنی و ایجاد فعالیت الکتریکی عضلانی با کاربرد تمرین ذهنی، بیانگر این نکته هستند که تمرین ذهنی از همان اصول تمرین فیزیکی تبعیت می کند. پس همان گونه که تمرین فیزیکی سبب افزایش هماهنگی حرکتی بیماران ضایعه مغزی می شود، می توان انتظار داشت کاربرد تمرین ذهنی هم منجر به بهبود هماهنگی این دوره را بیماران شود. هدف از مطالعه حاضر، بررسی تأثیر تمرین ذهنی روی هماهنگی حرکتی اندام فوقانی بیماران همی پلژیک بود. روش بررسی: پانزده بیمار همی پلؤپژیک (با دامنه سنی 70-20 سال) در این مطالعه شرکت داده شدند این بیماران مبتلا به آپراکسی و آفازیابودن همچنین از نظر شناختی مشکلی نداشتند بیماران به صورت تصادفی به سه گروه مساوی (تمرین ذهنی، تمرین فیزیکی  و تمرین ذهنی-فیزیکی) تقسیم شدند. هماهنگی حرکتی کلیه بیماران قبل و بعد از درمان همچنین یک ماه بعد از درمان مورد ارزیابی قرار گرفت. یافته ها: نتایج نشان داد که تمرین ذهنی و تمرین فیزیکی به طور معنی داری سبب بهبود هماهنگی حرکتی بیماران ضایعه مغزی می شود. بین گروه تمرین ذهنی و گروه تمرین فیزیکی اختلاف معنی داری مشاهد نشد و بالاخره اینکه کاربرد توام تمرین ذهنی و تمرین فیزیکی ثمربخش تر از زمانی بود که تنها یکی از این دو مورد استفاده قرار می گرفت. نتیجه گیری: از نتایج این مطالعه می توان اینطور نتیجه گرفت که تمرین ذهنی را می توان در امر توانبخشی بیماران مورد استفاده قرار داد.

Title: بررسی مقدار کاهش بین دو گوشی حاصله از گوشی داخلی تهیه شده از رسیور سمعک جیبی و مقایسه آن با گوشی استاندارد

http://rehabilitationj.uswr.ac.ir/article-1-512-fa.html

هدف: استفاده از پوشش بالینی در برخی مواقع تنها راه رسیدن به نتایج حقیقی آزمونهای شنوایی است که البته گاهی انجام آن با روشها و وسایل معمول میسر نمی باشد. در این راستا، روشهای مختلفی برای حل معضلات پوشش پیشنهاد شده که بهترین آن را می توان استفاده از گوشی های داخلی (به خاطر افزایش مقدار کاهش بین دو گوشی) ذکر کرد. روش بررسی: در پژوهش حاضر که در دپارتمان شنوایی شناسی دانشکده علوم توانبخشی دانشگاه علوم پزشکی ایران صورت گرفت، از رسیور سمعک جیبی مدل 21-22 viennaton ME برای ساخت گوشی داخلی استفاده شد و با مقایسه شبه تجربی میانگین کاهش بین دو گوشی (IA) حاصل از آن و گوشی مرسوم ادیومتر 822 OB بر روی 40 دانشجوی مذکر سنین 20 تا 25 سال، صورت گرفت. یافته ها: در روند بررسی ها مشخص گردید که مقدار (IA) در فرکانس 250،500،1000،2000 هرتز در گوشی داخلی بیشتر از گوشی مرسوم بوده، اما در فرکانس 4000 هرتز بازدهی گوشی داخلی کاهش یافته و منوط به محدودیت حداکثر شدت خروجی ادیومتر، مقدار IA قابل ارزیابی نیست. نتیجه گیری: از آنجا که گیرنده درج شده به آسانی قابل دسترس و آسان تر از مدل گوشی های معمولی است، این مدل از طرف شنوایی سنج ها توصیه می شود.

Title: بررسی ارتباط آگاهی واج شناختی و خواندن در کودکان 5/5 ساله و 5/6 ساله

http://rehabilitationj.uswr.ac.ir/article-1-513-fa.html

هدف: هدف پژوهش حاضر، آگاهی واج شناسی کودکان ، قبل از آموزش خواندن و در ضمن آموزش خواندن است تا مشخص نماید کدام یک از مولفه های آگاهی واج شناختی تحت تاثیر خواندن و کدام یک بدون تاثیر آن، در کودکان به وجود می آید. روش بررسی: برای بررسی آگاهی واج شناختی از آزمونی که بر اساس آزمون نورث و پارکر (1993) برنان و ارسون(1997) طراحی گردیده، استفاده شده است. این آزمون شامل سه بخش آگاهی هجایی، آگاهی واحدهای درون هجایی و آگاهی واجی است. آگاهی هجایی شامل دو تکلیف ترکیب و تقطیع هجا، آگاهی واحدهای درون هجایی شامل دو تکلیف تجانس و قافیه، آگاهی واجی شامل تکالیف نامیدن و حذف واج آغازین، شناسایی کلمات مختلف با واج آغازین یکسان، نامیدن و حذف واج انتهایی، شناسایی کلمات مختلف با واج انتهایی یکسان ، حذف واج میانی، تقطیع و ترکیب واجی است. آزمون روی یک گروه کودکان 5/5ساله و دو گروه کودکان 6/5 ساله انجام شده است. از دو گروه کودکان 6/5 ساله، یک گروه تحت آموزش خواندن قرار گرفته اند و گروه دیگر با کودکان 5/5 ساله در آمادگی حضور دارند. آزمودنی ها از کلاس های پایه اول ابتدایی و آمادگی 5 منطقه شمال، جنوب، شرق، غرب و مرکز شهر تهران با استفاده از روش نمونه گیری تصادفی انتخاب شدند. سپس از نظر هوشی رفتاری، شنوایی و زبانی بررسی گردیدند و در صورت عدم وجود مشکل در جنبه‌های بالا، از آنها آزمون آگاهی واج شناختی گرفته شد. یافته ها: یافته‌های پژوهش نشان می دهد که سه گروه آزمودنی، در میانگین امتیاز تکالیف مربوط به آگاهی هجایی و آگاهی واحدهای درون هجایی با یکدیگر تفاوتی ندارند. در تکالیف مربوط به آگاهی واجی، دو گروه 5/5 و 6/5 ساله ای که در مقطع آمادگی بوده اند و تحت آموزش رسمی خواندن قرار نگرفته اند، امتیاز پایینی به دست آوردند. میانگین امتیاز گروه 6/5 ساله ای که در پایه اول ابتدایی تحت آموزش خواندن قرار گرفته اند با دو گروه دیگر، در تمام تکالیف آگاهی واجی تفاوت دارد افزایش میانگین امتیاز آگاهی واجی در این  گروه ناشی از آموزش خواندن است. نتیجه گیری: بنابراین آگاهی واجی در بچه ها قبل بعد توانایی خواندن و هجی کردن حاصل می شود و آگاهی درون واجی در کودکان قبل از توانایی خواندن بوجود می آید.

Title: تعیین فراوانی علائم عصبی حسی و حرکتی در 309 بیمار دیابتی مراجعه کننده به مرکز دیابت همدان در سال 1378

http://rehabilitationj.uswr.ac.ir/article-1-514-fa.html

هدف: دیابت یک بیماری شایع است که در تمام جوامع دیده می شود. این بیماری، دستگاه های مختلف بدن از جمله سیستم عصبی را درگیر می کند. اصولا عوارض دیابت روی سیستم عصبی غیر قابل برگشت و ناتوان کننده است. در این مطالعه، بیشتر روی شناسایی علائم عصبی دیابت به خصوص عوارض حسی و حرکتی تلاش شده است، تا شاید با شناسایی آنها و مقایسه با آمارهای جهانی بتوان نسبت به تشخیص و کنترل سریع آن اقداماتی انجام داد. روش بررسی: در این مطالعه که یک مطالعه توصیفی است ، ابتدا 309 بیمار مبتلا به دیابت که در مرکز دیابت دانشگاه علوم پزشکی همدان دارای پرونده بودند، انتخاب شدند. براساس اطلاعات مورد نیاز، فرمی تهیه و با توجه به معاینه دقیق سیستم عصبی  برای هر بیمار تکمیل شده در نهایت ، اطلاعات جمع آوری شده مورد تجزیه و تحلیل آماری قرار گرفتند. یافته‌ها: در بررسی مشخص شد که بیشترین شکایت حسی، احساس گزگز و سوزش و بیشترین شکایت حرکتی، پارزی در اندامهاست. این عوارض بیشتر در گروه سنی 50 سال به بالا مشهود بود که بیش از 5 سال از زمان تشخیص دیابت آنها گذشته بود. نتبجه‌گیری: از آنجا که دیابت یک بیماری همراه با عوارض متعدد از جمله نوروپاتی است که می تواند باعث ناتوانی بیمار شود، شناسایی این عوارض و زمینه های ایجاد آن می تواند در جلوگیری از بروز آنها و در نهایت، ایجاد زندگی بهتر برای بیماران دیابتی کمک کننده باشد.

Title: بررسی بروز انواع صدمات ارتوپدی در 796 بیمار مراجعه کننده به درمانگاه اورژانس ارتوپدی بیمارستان سینا

http://rehabilitationj.uswr.ac.ir/article-1-515-fa.html

هدف: در کلیه مراکز درمانی براساس ارزیابی تعداد بیماران مراجعه کننده، نوع ضایعات و علل ایجاد آنها، نسبت به پیشگیری، درمان و توانبخشی ضایعات، اقدامات ضروری صورت می گیرد. درمانگاه ارتوپدی بیمارستان سینا به عنوان مرکز ارجاع تروما در شبانه روز پذیرای بیماران بسیاری از تهران و شهرستان ها است. در این مطالعه، تلاش می شود به بررسی توصیفی بیماران مراجعه کننده به درمانگاه ارتوپدی بیمارستان سینا و طبقه بندی آنها، در مورد زمینه شناسی برخی آسیبهای شایع پرداخته شود. روش بررسی: 796 بیمار طی ماه های آبان، آذر و دی 1377 به درمانگاه ارتوپدی بیمارستان سینا مراجعه کرده اند. متوسطه سنی بیماران 28/8 سال و %78 آنها مذکر هستند. 530 نفر از بیماران (%67) دچار انواع شکشتگی ، 49 نفر (%6) دچار انواع در رفتگی مفاصل ، 84 نفر (%10) دچار ضایعات له شدگی اندام و مابقی دچار صدمات لیگامانی شده اند. %30 بیماران در اثر زمین خوردن، %22 در اثر تصادف به صورت عابر پیاده، %5 در اثر تصادف با موتور سیکلت، دچار ضایعه شده اند. از این بیماران، 250 نفر نیاز به بستری و درمان در بیمارستان داشته اند. یافته ها: توجه دقیق به تعداد مراجعان به بیمارستانها و مراکز درمانی در تهران و شهرستانها و حتی در مناطق مختلف تهران و میزان توانایی آنها در ارائه خدمات با توجه به پرسنل تعداد تختهای موجود، وجود وسایل مصرفی بیماران ارتوپدی و تجهیزات، بسیار مهم می باشد  و مطالعه توصیفی حاضر وجود مشکل قابل بررسی جدی ای را نشان می دهد. نتیجه گیری: برنامه ریزی دقیق ایجاد مراکز درمانی با امکانات و قابلیتهای شایسته، بر اساس تعداد مراجعان و آمارهای توصیفی ضرورت دارد. این مطالعه شروع یک مطالعه گسترده در این زمینه است.

Title: بررسی شیوع سنی و جنسی صرع و عوامل موثر بر آن در بیماران مراجعه کننده به بیمارستانهای سینا و قائم همدان در سالهای 68 تا 74

http://rehabilitationj.uswr.ac.ir/article-1-516-fa.html

هدف: بیماری صرع به عنوان یکی از بیماریهای شایع نورولوژی ، در تمام جوامع از اهمیت خاص برخوردار است.  یکی از مواردی می توان در شناخت و درمان این بیماری کمک کننده باشد، شناخت شیوع بیماری و عواملی است که می تواند آنرا تحت تاثیر خود قرار دهد. در این مقاله ، بررسی شیوع سنی و جنسی صرع از اهداف اصلی بوده و همزمان، فاکتورهای موثر دیگر که بر سیر بیماری تاثیر داشته اند، بررسی شده است. روش بررسی: این مطالعه ، یک مطالعه توصیفی گذشته نگر مربوط به سالهای بین 1368-74 است و با استفاده از 290 پرونده بیمارانی که با تشخیص اپی لپسی بستری شده بودند ، انجام گرفت. پس از تهیه پرسشنامه، اطلاعات مورد نیاز جمع آوری و متعاقب آن، با بررسی آمارهای مربوطه، جداول و نمودارها ترسیم شدند. یافته‌ها : بیماری صرع در مردان شایع تر از زنان و شایع ترین محدوده سنی مراجعه کننده سن 11-20 سالگی بود. شایعترین سن شروع بیماری  2-10 سالگی بود. در بین عوامل مؤثر بر آن، بیماری های مغزی، تب و تشنج  شایع تر از عوامل دیگر بوده است. نتیجه‌گیری: از آنجا که صرع یک بیماری با درمان طولانی است شناخت زمینه های ایجاد آن و پیشگیری و یا بهبود عوامل موثر بر آن می تواند در درمان موفق بیماری کمک کننده باشد.

Title: آخرین یافته های علمی در زمینه اتیولوژی اوتیسم

http://rehabilitationj.uswr.ac.ir/article-1-519-fa.html

اوتیسم و سایر ضایعات در طیف اوتیسم که به عنوان سندرم های رفتاری تعریف شده اند ، می تواند یک ناتوانی طولانی مدت باشد. علت اصلی کاملا شناختهشده نیست ، اما تحقیقات اخیر ، گویایی یفته های جدیدی در مورد علت اوتیسم هستند و مقاله حاضر ، خلاصه ای از این تحقیقات را ارائه می دهد. مواردی که در اتیولوژی اوتیسم مطح هستند ، عبارتند از : منشا نورو بیولوژیکال ، تحقیقات نورو بیولوژیکال نشان می دهند که سیستم های دوپامین و سورتونی نیز در پاتوژنزا اوتیسم نقش دارند. ژنتیک ، نقش فکتورهای ژنتیکی ، با مطالعات روی کروموزوم X و همین طور در دوقلوهای یک تخمی (منوزیگوت ) مشخص شده است. بررسی متابولیسم کلوکز مغزی در اوتیسم اولیه ، در بیماران اوتیستیک ، آسیمتری جیروس قدامی به صورت راست < چپ نشان داده شده است و این برخلاف وضعیت نرمال راست >چپ است. بررسی جریان خون منطقه ای مغزی ، در اتیسم الیه ، گویای عدم اختلال عملکرد منطقه ای کورتیکال می باشد. بررسی همراهی اوتیسم با اپی لپسی ، مشخص می کند که اپی لپسی در بیماران اوتیستیک منجر به پس رفت در زبان رفتارهای اجتماعی و شناختی می شود. بررسی همراهی اوتیسم با توبروس اسکلروزیس ، نشان می دهد که در این بیماران توبرهای مغزی نسبت به بقیه بیماران توبروس اسکلروزیس بیشتر است. منشاء آمبریولوژیکال برای اوتیسم ، گویای آنومالیهای تکاملی هسته های حرکتی اعصاب کرانیال می باشد. نقش عوارض زایمانی در اوتیسم ، که در گذشته به عنوان اتیولوژی اوتیسم مطرح می شد در تحقیقات جدید تایی می شود مطالعه نقش آلرژی غذایی در اوتیسم ، مشخص کننده بدتر شدن علائم نورولوژیکال در بیماران اوتیستیکی است که مبتلا به آلرژی غذایی بعد از مصرف شیر و گندم هستند. در اندازه گیری دور سر بچه های اوتیستیک ، افزایش دور سر و افزایش رشد در این بچه ها نسبت به سایر کودکان مشهود است.

Title: ضرورت ساخت/هنجاریابی مقیاس رفتار انطباقی در تشخیص، آموزش، درمان و توانبخشی افراد و ارزیابی برنامه ها

http://rehabilitationj.uswr.ac.ir/article-1-520-fa.html

رفتار انطباقی را به عنواون روشی که مردم به وسیله آن با خواسته های طبیعی و اجتماعی خود کنار می آیند تعریف می کنند. (1) اختلالات رفتار انطباقی این گونه تشریح شده اند: محدودیت های قابل ملاحظه در کارآیی فرد برای رسیدن به معیارهای رسش، یادگیری، استقلال شخصی و مسئولیت اجتماعی که بر اساس سنجش بالینی و مقیاس های استاندارد شده، با توجه به گروه سنی و فرهنگی فرد، از وی انتظار می رود. (2) تعریف رفتار انطباقی و توصیف نارسایی های سازگاری بر توانایی فرد در کنار آمددن با خواسته های محیط خود دلالت دارد و برخی اندیشمندان، زمانی که رابطه رفتار انطباقی را با کم توانی ذهنی شرح می دهند، از این عقیده حمایت می کنند. (2) علی رغم این واقعیت که مقیاس های رفتار انطباقی از ابزارهای ضروری در تشخیص، آموزش، درمان و توانبخشی افراد (بویژه افراد مبتلا به اختلالات رشدی) و ارزیابی برنامه ها هستند، افراد متخصص به آنها دسترسی ندارند. این مقاله سعی دارد ضرورت ساخت/هنجاریابی چنین مقیاس هایی را توضیح داده، نمونه ای از مشهورترین آنها را با عنوان "مقیاس رفتار انطباق-اقامتی-اجتماع" معرفی نماید.

Title: اختلالات هویت جنسی دوران کودکی

http://rehabilitationj.uswr.ac.ir/article-1-521-fa.html

هویت جنسی عبارت است از احساس فردی مرد یا زن بودن که از سنین 3 و 4 شروع می شود. برای تشخیص اختلال هویت جنسی در DSMIV دو ملاک عمده وجود دارد که ملاک الف – همانند سازی قوی و مستمر با جنس مخالف و ملاک ب – اساس ناراحتی مستمر با جنس خود یا احساس نامتناسب بودن در نقش جنسی خود را شامل می شود. شیوع این اختلال ، از %6-16 در مطالعات همه گیر شناسی غیر رسمی متغیر بوده است. برای ارزیابی این اختلال می توان از گزارش والدین، روشهای رفتاری و فنون فرافکن استفاده کرد. از عارضه های همراه این اختلال می توان به افسردگی، آشفتگیهای رفتاری و اختلالات شخصیت اشاره کرد. بین سوگیری جنسی و هویت جنسی رابطه قطعی  نمی توان فرض کرد. در مدل نظری زاکر برادی و کواتیس، یک عامل عمومی که اضطراب کودک را افزایش داده و عوامل اختصاصی که به صورت پویا در درون خانواده یا کودک وجود دارند، به عنوان علل اصلی اختلال هویت جنسی معرفی شدند. برای درمان می توان از فنون رفتاری و روان تحلیلی و آموزش خانواده استفاده کرد، ولی کار با خود بیمار و درمان عارضه های همراه از جمله افسردگی و سایر مشکلات روانی–اجتماعی، از اهمیت ویژه ای برخوردار است.

Title: گزارش موردی: گزارش یک مورد اختلال راه رفتن به علت متیل مالونیک اسیدوری همراه هموسیستینوری

http://rehabilitationj.uswr.ac.ir/article-1-522-fa.html

این اختلال به قدری نادر است که تا کنون تنها 100 مورد در دنیا گزارش شده است. در این اختلال ، آنزیم کبدی (متیل مالونیل کوآموتاز ) مسئول انجام یکی از چند صد فرایند شیمیایی که عهده دار تبدیل پروتئین به انرژی یا سایر مواد هستند، دچار اشکال است. تظاهرات بالین این اختلال در ماههای اول زندگی ممکن است به صورت کاهش رشد لتارژی ، امتناع از خوردن، عقب ماندگی ذهنی و تشنج باشد. تظاهرات دیررس شامل سایر اختلالات عصبی مانند دمانس ، میلوپاتی و اختلال راه رفتن است. افزایش سطح اسیدمتیل مالونیک و هموسیستین پلاسما تشخیص را مسجل می سازد. برخلاف بیماران هموسیستینوری کلاسیک، سطح متیونین پلاسما در این بیماران طبیعی است. بیمار دختر 13/5 ساله ای است که به علت بروز تشنج و از دست دادن توانایی راه رفتن، از دست دادن اشتها، بروز بی اختیاری ادراری و پسرفت هوشی مراجعه نمود. در بررسیهای به عمل آمده سطوح اسیدهای متیل مالونیک و هموسیستین پلاسما و ادرار شدیدا بالا بود. بیمار با دوز بالای ویتامین B12  ، بتائین و کربامازپین و همچنین فیزیوتراپی تحت درمان قرار گرفت. این درمان، سبب بهبودی واضح در وضعیت عصی و ذهنی شد. متیل مالونیک اسیدمی و هموسیتینوری نیز کنترل شد.
